# ChatGPT’s Performance on Portuguese Medical Examination Questions: Comparative Analysis of ChatGPT-3.5 Turbo and ChatGPT-4o Mini

**DOI:** 10.2196/65108

**Published:** 2025-03-05

**Authors:** Filipe Prazeres

**Affiliations:** 1 Faculty of Health Sciences, University of Beira Interior, Av. Infante D. Henrique, Covilhã, 6201-506, Portugal, 351 234393150; 2 Family Health Unit Beira Ria, Gafanha da Nazaré, Portugal; 3 CINTESIS@RISE, Department of Community Medicine, Information and Health Decision Sciences, Faculty of Medicine of the University of Porto, Porto, Portugal

**Keywords:** ChatGPT-3.5 Turbo, ChatGPT-4o mini, medical examination, European Portuguese, AI performance evaluation, Portuguese, evaluation, medical examination questions, examination question, chatbot, ChatGPT, model, artificial intelligence, AI, GPT, LLM, NLP, natural language processing, machine learning, large language model

## Abstract

**Background:**

Advancements in ChatGPT are transforming medical education by providing new tools for assessment and learning, potentially enhancing evaluations for doctors and improving instructional effectiveness.

**Objective:**

This study evaluates the performance and consistency of ChatGPT-3.5 Turbo and ChatGPT-4o mini in solving European Portuguese medical examination questions (2023 National Examination for Access to Specialized Training; Prova Nacional de Acesso à Formação Especializada [PNA]) and compares their performance to human candidates.

**Methods:**

ChatGPT-3.5 Turbo was tested on the first part of the examination (74 questions) on July 18, 2024, and ChatGPT-4o mini on the second part (74 questions) on July 19, 2024. Each model generated an answer using its natural language processing capabilities. To test consistency, each model was asked, “Are you sure?” after providing an answer. Differences between the first and second responses of each model were analyzed using the McNemar test with continuity correction. A single-parameter *t* test compared the models’ performance to human candidates. Frequencies and percentages were used for categorical variables, and means and CIs for numerical variables. Statistical significance was set at *P*<.05.

**Results:**

ChatGPT-4o mini achieved an accuracy rate of 65% (48/74) on the 2023 PNA examination, surpassing ChatGPT-3.5 Turbo. ChatGPT-4o mini outperformed medical candidates, while ChatGPT-3.5 Turbo had a more moderate performance.

**Conclusions:**

This study highlights the advancements and potential of ChatGPT models in medical education, emphasizing the need for careful implementation with teacher oversight and further research.

## Introduction

Generative artificial intelligence (AI) represents a branch of AI dedicated to the development of systems that can autonomously generate high-quality digital content on demand, and it can do so across various modalities, such as written text, images, audio, and video [1-3]. Generative AI tools are trained on large datasets, enabling them to produce work that mirrors human-created content [2]. Nowadays, there are several examples of generative AI tools, including ChatGPT (OpenAI Inc), Runway, Gemini (Google Inc), DALL-E (OpenAI Inc), Copilot (Microsoft Inc), Midjourney, NovelAI (Anlatan), Claude (Anthropic), and Jasper AI, among others. ChatGPT, the large language model (LLM) chatbot, developed by OpenAI [4], that started the AI boom in November 2022, became the most popular AI tool of 2023, accounting for over 60.2% of visits between September 2022 and August 2023, with a total of 14.6 billion website visits [5]. ChatGPT’s availability as a free-to-use, low-bandwidth service may reduce disparities compared to paid versions or models by making advanced AI technology accessible to a broader and more diverse global population [6], contributing to making it the most popular generative AI tool [7].

Recent literature reviews regarding AI have shown that this type of technology has potential applications in several fields, spanning from the architecture, engineering, and construction industry to health care [8-11]. The possible applications in medicine are substantial, ranging from diagnostic and treatment support (eg, clinical imaging improvement, classification of diseases, prediction of disease onset, development of treatment, and medication prescriptions) [12] to facilitate communication and engagement between medical professionals and their patients [13], and also improving medical education and its accessibility [10,14,15]. For example, ChatGPT can be used as a study tool to clearly explain complex medical concepts [16,17] (eg, radiology reports [18]), create memory aids for challenging topics, clarify medical practice questions, summarize research articles, compile lists of differential diagnoses [17], generate medical examination questions [19], and simulate physician-patient interactions [14].

Medical written examinations are an important part in evaluating the competence and knowledge of medical students and graduates (eg, access of physicians to specialized training, such is the case in Portugal). These examinations not only test factual knowledge but also evaluate the critical thinking and problem-solving skills of the candidates. With the recent growing interest in AI, an important question arises: Can AI, specifically ChatGPT, perform at a level comparable to human candidates in medical written examinations? By evaluating ChatGPT’s ability to correctly answer medical questions, its medical proficiency and its potential role as an educational tool can be assessed. Successfully completing this task can demonstrate ChatGPT’s capability to serve as a resource for medical students by providing continuous access to information, particularly benefiting students in remote or under-resourced areas [6].

ChatGPT is known for having the capability of performing near the passing threshold of 60% accuracy of the United States Medical Licensing Examination (USMLE) [20] and for approximately having the knowledge equivalent to a third-year medical student [21]. ChatGPT’s performance on medical examinations has been analyzed across different countries and questions. A 2023 systematic review with a meta-analysis of 19 articles found a mean performance of ChatGPT of around 61% [22], and a more recent review published in 2024 concluded that, despite ChatGPT’s satisfactory performance in examinations, further studies are necessary to fully explore its potential in medical education [23].

Furthermore, ChatGPT struggles with non-English language assessments possibly due to a limited understanding of linguistic nuances and Western-centric internet data, which may not fully represent the clinical and disease differences in some countries, like African and Asian populations [24], warranting more studies in other languages to ensure better understanding of ChatGPT’s accuracy in diverse cultural contexts. For example, ChatGPT performed considerably lower on a medical examination in Chinese (45.8% correct answers on the Chinese National Medical Licensing Examination) [25], and even worse in the French examination with 22% correct answers [26].

In July 2024, OpenAI launched GPT-4o mini, a smaller version of its latest GPT-4o (“o” for “omni”) AI language model. This new model replaced GPT-3.5 Turbo in ChatGPT, making this an ideal time to study the performance of both free models in resolving written medical examinations.

This study aims to evaluate the performance and consistency of 2 AI models, ChatGPT-3.5 Turbo and ChatGPT-4o mini, in solving the questions of a non-English language (European Portuguese) written medical examination, with a format of multiple-choice with one best answer—the 2023 National Examination for Access to Specialized Training (Prova Nacional de Acesso à Formação Especializada [PNA])—and compare their performance to that of human candidates.

## Methods

### Study Design

The PNA examination is part of the requirements for entering specialized medical training in Portugal. Its purpose is to rank candidates for accessing specialized training vacancies, so no minimum passing grade is needed [27].

The PNA questions used in this study were from the actual 2023 Portuguese PNA examination, which is publicly available on the web [27]. This examination includes 150 questions with 5 multiple-choice answers each, with only a single best answer, similar to the USMLE. The questions are based on clinical vignettes and divided into 2 parts with 75 questions each. The examination emphasizes clinical reasoning and the application and integration of clinical knowledge and is scored on a scale from 0 to 150 points, with no penalties for blank or incorrect answers. It covers various medical disciplines, including medicine, surgery, pediatrics, gynecology and obstetrics, and psychiatry. The examination duration is 240 minutes, divided into 2 parts of 120 minutes each [27].

ChatGPT-3.5 Turbo was provided with the first part of the examination (74 no image-based multiple-choice questions [MCQs]) on July 18, 2024, and ChatGPT-4o mini with the second part of the examination (74 no image-based MCQs) on July 19, 2024. The questions were entered into the models in European Portuguese and in a format similar to how they are presented to human candidates, and each model was requested to provide a single-letter answer, just like human candidates. For each question, the models generated an answer using their natural language processing capabilities. Following each model’s response, a follow-up question, “Are you sure?” was asked to test for consistency—this technique was previously used by Brin et al [28]. An example of the input format of the questions and the respective responses by ChatGPT in European Portuguese is depicted in Table 1, with corresponding translations to English performed by ChatGPT-4o mini. Each question was addressed in a new chat session to reduce the potential influence of memory retention bias of ChatGPT.

**Table 1.** Example of the input format of the questions and the respective responses by ChatGPT.

	Examination question in European Portuguese	Examination question translated to English (performed by ChatGPT-4o mini)
Question	Um homem de 73 anos vem à consulta hospitalar para reavaliação de doença pulmonar obstrutiva crónica. Na consulta prévia, há seis meses, apresentava-se em estadio GOLD B. Refere agora, desde há três meses, agravamento da dispneia para esforços médios, sem alteração do padrão habitual de tosse ou de expetoração. Nega febre, perda de apetite ou outras queixas de novo. A história médica revela ainda obesidade. A medicação habitual inclui brometo de tiotrópio e salmeterol. É ex-fumador de 40 UMA desde há 10 anos. Os sinais vitais são temperatura 36 °C, frequência respiratória 18/min, frequência cardíaca 78/min e pressão arterial 115/89 mm Hg; SpO2 94% (ar ambiente). Ao exame físico apresenta cianose labial, com aparência confortável e atrofia muscular na área temporal. A auscultação pulmonar revela crepitações raras dispersas, sem prolongamento do tempo expiratório. O restante exame físico, incluindo a auscultação cardíaca, encontra-se dentro dos parâmetros de normalidade. Qual das seguintes alternativas mais provavelmente reduz o risco de morte relacionada com esta doença? Azitromicina. Montelucaste. Oxigenoterapia. Prednisolona. Teofilina. Indique apenas a letra da resposta, sem justificação.	A 73-year-old man comes to the hospital for reevaluation of chronic obstructive pulmonary disease (COPD). At the previous visit six months ago, he was in GOLD stage B. He now reports worsening shortness of breath with moderate exertion over the past three months, without changes in his usual cough or sputum production. He denies fever, loss of appetite, or any new complaints. His medical history also reveals obesity. His usual medications include tiotropium bromide and salmeterol. He is a former smoker with a 40 pack-year history, quit 10 years ago. Vital signs are temperature 36 °C, respiratory rate 18/min, heart rate 78/min, and blood pressure 115/89 mm Hg; SpO2 94% (room air). On physical examination, he shows lip cyanosis, appears comfortable, and has muscle atrophy in the temporal area. Lung auscultation reveals scattered rare crackles, with no prolonged expiratory phase. The rest of the physical examination, including cardiac auscultation, is within normal limits. Which of the following is most likely to reduce the risk of death related to this condition? Azithromycin. Montelukast. Oxygen therapy. Prednisolone. Theophylline. Please indicate only the letter of the answer, without justification.
ChatGPT-3.5 Turbo answer	A resposta correta é: (C) Oxigenoterapia.	The correct answer is: (C) Oxygen therapy.
Follow-up question	Tem a certeza?	Are you sure?
ChatGPT-3.5 Turbo answer	Peço desculpa pelo erro anterior. Vamos rever a situação: Dadas as características do paciente com DPOC grave e hipoxemia (SpO2 de 94% em ar ambiente), a intervenção que mais provavelmente reduzirá o risco de morte relacionada com esta doença é a oxigenoterapia. Portanto, a resposta correta é: (C) Oxigenoterapia.	I apologize for the previous error. Let’s review the situation: Given the characteristics of the patient with severe COPD and hypoxemia (SpO2 of 94% on room air), the intervention that is most likely to reduce the risk of death related to this condition is oxygen therapy. Therefore, the correct answer is: (C) Oxygen therapy.

Human candidates’ scores were obtained from the official records of the PNA 2023 examination, which are also publicly available on the web [27].

To compare the mean scores of human candidates to those of the language models, all questions of the PNA 2023 examination had to be answered. Since the examination included 2 questions using images (one in the first part and another one in the second part; both with electrocardiogram strips), these questions were answered by GPT-4o, as it can handle images in addition to text.

### Ethical Considerations

This study exclusively used data that had been previously published online and did not involve direct interaction with human participants. As a result, ethical guidelines pertaining to human participants are not applicable.

### Statistical Analysis

Analyses were performed using IBM SPSS Statistics (Version 21). The McNemar test [29] with continuity correction [30] was used to determine differences between the first and second responses of ChatGPT-3.5 Turbo and ChatGPT-4o mini. Single-parameter *t* test was used to compare the performance of ChatGPT-3.5 Turbo and ChatGPT-4o mini with that of human candidates. Frequencies and percentages were used for categorical variables and means and CIs for numerical variables. Statistical significance was considered at *P*<.05.

## Results

### Overall Performance and Consistency

In the initial response with ChatGPT-3.5 Turbo, of the 74 questions, 40 (54%) answers were correct and 34 (46%) answers were incorrect. After the follow-up question, “Are you sure?,” the number of correct answers decreased to 28 (38%), while the number of incorrect answers increased to 46 (62%). This change occurred because ChatGPT-3.5 Turbo corrected 12 originally incorrect answers, but also changed 24 originally correct answers to incorrect. This pattern of change approached, but did not reach, significance (*χ*²_1_=3.361, *P*=.067).

Initially, of the 74 questions, ChatGPT-4o mini produced 48 (65%) correct answers and 26 (35%) incorrect answers. After being asked, “Are you sure?,” the correct answers dropped to 42 (57%), while incorrect answers rose to 32 (43%). This change occurred because ChatGPT-4o mini fixed 12 previously wrong answers but also changed 18 previously correct answers to incorrect. This pattern of change was not statistically significant (*χ*²_1_=0.833, *P*=.361).

The 2 questions using images (one in the first part and another one in the second part) were answered correctly by GPT-4o.

### LLM Chatbot Versus Human

When evaluating AI capabilities in relation to human abilities, LLM responses in part 1 of PNA (74 questions resolved by ChatGPT-3.5 Turbo plus 1 by GPT-4o) showed lower accuracy than human respondents. The human mean score was statistically significantly higher by 6.04 (95% CI 5.65-6.43) than the LLM score of 41 (*P*<.001).

In part 2 of PNA (74 questions resolved by ChatGPT-4o mini added to 1 question by GPT-4o), the LLM score showed higher accuracy than human respondents. The human mean score was statistically significantly lower by 5.58 (95% CI 5.25-5.9) than the LLM score of 49 (*P*<.001).

## Discussion

### Principal Findings

This study analyzes the performance of 2 ChatGPT models (ChatGPT-3.5 Turbo and ChatGPT-4o mini) on the Portuguese medical written examination: 2023 National Examination for Access to Specialized Training, revealing important differences in accuracy and consistency. Although, both ChatGPT-3.5 Turbo and ChatGPT-4o mini answered correctly in the majority of the questions, ChatGPT-4o mini achieved a higher accuracy rate of 65% (48/74) compared to ChatGPT-3.5 Turbo’s 54% (40/74), demonstrating a superior capability in handling medical questions. Additionally, ChatGPT-4o mini showed greater consistency in confirming answers, highlighting its reliability. When evaluated against human respondents, ChatGPT-4o mini outperformed the average human accuracy, while ChatGPT-3.5 Turbo fell short.

### Strengths

This study stands out for its innovative approach in analyzing the performance of ChatGPT-3.5 Turbo and ChatGPT-4o mini in a medical examination context. It is the first to evaluate these models using an examination conducted in a less commonly studied language, Portuguese, thereby broadening the scope of language-specific AI assessments. By incorporating the actual scores of human candidates for comparison, the study provides a robust benchmark against real-world performance. Furthermore, the research examines the stability of the AI’s answers by repeatedly asking “Are you sure?,” offering valuable insights into the consistency of the responses.

### Comparison to Prior Work

A recent study evaluated ChatGPT’s performance on medical licensing examinations across multiple countries (United States, Italy, France, Spain, United Kingdom, and India) and determined a variable accuracy, ranging from 22% on the French examination to 73% on the Italian examination [26]. In this study, ChatGPT answered correctly in more than 50% of the Portuguese medical examination questions, positioning it next to the countries with better performance. For example, in a Turkish study, ChatGPT reached 70.9% accuracy in the medical specialty examination [31]. In the Iranian medical licensing examination, ChatGPT performed with 68.5% of the questions answered correctly [32]. And in Poland, ChatGPT achieved a 67.1% correct response rate on the Polish medical specialization licensing examination [33].

When analyzing the differences between the 2 ChatGPT versions, ChatGPT-4o mini outperformed ChatGPT-3.5 Turbo in this study: 65% (48/74) vs 54% (40/74) correct response rate. This suggests that advancements in the underlying architecture and training data of ChatGPT-4o mini (knowledge up to October 2023) have improved its capability to understand and respond to medical questions with more accuracy. Previous studies evaluating the performance of different ChatGPT models found that ChatGPT-4 consistently performed better compared to ChatGPT-3.5. For example, ChatGPT-4 outperformed ChatGPT-3.5 on the Polish Medical Final Examination [34], the Spanish Medical Residency Entrance Examination (Médico Interno Residente) [35], the 2023 Japanese Nursing Examination [36], the Peruvian National Licensing Medical Examination (Examen Nacional de Medicina) [37], and in the USMLE soft skill assessments [28], to name a few. Nonetheless, ChatGPT-4 is a paid model and thus not accessible to everyone, which is not the case for the most recent free-to-use ChatGPT-4o mini.

Another important aspect is consistency. The results of this study revealed that ChatGPT-3.5 Turbo was less stable when asked to confirm its original answers. These results are consistent with those of Brin et al [28], who found that ChatGPT-3.5 altered its answers 82.5% of the time in the USMLE assessments [28]. Unfortunately, in this study, it was not shown that by changing the original answers, ChatGPT-3.5 Turbo improves its accuracy. This contrasts with studies on human students, which have shown that changing their answers usually improves their test scores [38]. One can wonder, since the “awareness of what one knows and does not know depends in part on how much one knows” [39], does ChatGPT-3.5 Turbo change its answers because it does not know, or does it simply change answers to satisfy the user when prompted?

When evaluating the AI models against human respondents, it was found that in part 2 of the PNA examination (74 questions resolved by ChatGPT-4o mini plus 1 question by GPT-4o), the LLM outperformed the average accuracy of human participants. In contrast, in part 1 of the PNA examination (74 questions resolved by ChatGPT-3.5 Turbo plus 1 question by GPT-4o), LLM showed lower accuracy than human respondents. This indicates that while earlier versions, like ChatGPT-3.5 Turbo, may have required a high degree of human oversight, more recent and advanced versions, like ChatGPT-4o mini, have the potential to match or exceed human performance in medical domains. Although no previous studies have analyzed the performance of ChatGPT-4o mini, and no direct comparisons can be made, some studies have already noted that LLMs outperformed human candidates in several medical examinationinations (eg, the German Medical State Examinations of 2022 [40], part 1 of the Fellowship of the Royal College of Ophthalmologists MCQ examination [41], and the University of Toronto Family Medicine Residency Progress Test [42]).

### Limitations

This study has several limitations regarding the performance evaluation of ChatGPT-3.5 Turbo and ChatGPT-4o mini. The analysis was based solely on ChatGPT’s indication of the correct answer, which, while aligning with expectations for human candidates, does not consider other aspects of examination performance. Additionally, the grading did not account for the complexity or length of the questions, providing an incomplete assessment of the models’ performance. Further studies should incorporate a more comprehensive evaluation framework that considers the reasoning process and evaluates performance across a broader range of question types and difficulties.

### Future Perspectives

This study highlights the importance of continuous improvement in ChatGPT models to further enhance their reliability and accuracy. The superior performance of ChatGPT-4o mini compared to its predecessor offers promising applications in medical education. Its higher accuracy and consistency suggest that it could serve as an effective tool for training medical students. However, a broader assessment of ChatGPT-4o mini across various tests and real-world scenarios is required, as good performance on a specific test may not indicate abilities for general and reliable medical education usage. Additionally, there are known drawbacks and ethical considerations when using AI applications, including the potential for fabricated, incorrect, or biased information [43]. Other issues include limited training periods and the possibility of providing different answers to the same question depending on how the question is phrased [43]. A recent systematic scoping review by Xu et al [44] advises medical students to use ChatGPT cautiously, cross-checking information with reliable sources and disclosing AI-generated content in their work. Teachers should guide students on the effective and ethical use of ChatGPT, assess its reliability, and explore mixed assessment methods to evaluate student abilities while considering its impact on traditional assignments [44].

### Conclusion

On the 2023 Portuguese National Examination for Access to Specialized Training, ChatGPT-4o mini achieved an accuracy rate of 65% (48/74), surpassing ChatGPT-3.5 Turbo. This demonstrates a superior capability in handling medical questions. ChatGPT-4o mini outperformed medical candidates, while ChatGPT-3.5 Turbo had a more moderate performance. This study highlights the advancements and potential of ChatGPT models in medical education, emphasizing the importance of careful implementation with teacher oversight and further research.

## Abbreviations

AI: artificial intelligence
LLM: large language model
MCQ: multiple-choice question
PNA: Prova Nacional de Acesso à Formação Especializada
USMLE: United States Medical Licensing Examination
